# Children's Positive and Negative Emotional Responses to an HIV Disclosure Study in South Africa

**DOI:** 10.3389/fped.2022.857336

**Published:** 2022-05-31

**Authors:** Candice W. Ramsammy, Lisa Galvin, Celeste Joyce, Given Leshabane, Janice Buckley, Kennedy Otwombe, Afaaf Liberty, Avy Violari

**Affiliations:** ^1^Perinatal HIV Research Unit, Faculty of Health Sciences, University of the Witwatersrand, Johannesburg, South Africa; ^2^Department of Psychiatry, Faculty of Health Sciences, School of Clinical Medicine, University of the Witwatersrand, Johannesburg, South Africa; ^3^Faculty of Health Sciences, School of Public Health, University of the Witwatersrand, Johannesburg, South Africa

**Keywords:** disclosure, perinatal infection, human immunodeficiency virus (HIV), pediatric model, chronic illness, emotional response, internalizing and externalizing behavior

## Abstract

The benefits of HIV status disclosure to children is widely cited. However, few studies have reported how children respond to the process in a longitudinal fashion. This paper describes children's responses, as documented by healthcare providers (HCPs), during a longitudinal disclosure study conducted at Chris Hani Baragwanath Academic Hospital in Soweto, South Africa. Two HCPs facilitating disclosure recorded observations of 30 participating children (60% female), aged 7-13 years. Participants attended an average of six disclosure counseling sessions over 78 weeks. Observations documented by HCPs included the child's behavior and expressed emotions during the disclosure counseling sessions. The data was analyzed using content analysis. Mixed responses were observed in children who received full disclosure (27/30), with more children responding with strong negative emotions (16/27). However, 10 of those responded well to reassurance, and emotionally improved over subsequent sessions. Improvements were also observed in the communication and relationship between caregivers and children (17/30). Although most children understood the disclosure content (17/30), many were avoidant of the topic of HIV (16/30). With the understanding of the complex range of emotions elicited by HIV disclosure, we can better prepare HCPs on what to anticipate and train caregivers to further manage negative responses post-disclosure. This in turn may lead to more positive experiences of disclosure and the child's healthy acceptance of their HIV status.

## Introduction

Disclosure of HIV status has well documented benefits, including better anti-retroviral treatment (ART) adherence (1–4), improved behavior, school attendance, school performance, social functioning, as well as reduced stress (5). Disclosure may allow individuals to express their feelings and thoughts, develop their sense of self, and build stronger interpersonal relationships (6). As children approach adolescence and sexual debut, disclosure is important in encouraging safe sex practices, and reducing the number of horizontal transmissions (2).

Despite the benefits, disclosure of HIV status to children remains low in sub-Saharan Africa. In South Africa, between 9 and 40% of children living with HIV (CHIV) had been disclosed to by ~10 years of age (7–9). Although there are interventions that support caregivers in disclosing their own HIV positive status to their children (10–12), few are in place that aid caregivers in disclosing the child's HIV status to them (13, 14). One pediatric HIV disclosure intervention developed in 2003 is now used as a guideline across Botswana (15), while another was introduced by the Namibian Ministry of Health and Social Services into their routine pediatric HIV service in 2010 (1). Not only do these interventions promote disclosure, but they also reduce caregiver stress and support the mental health of the child (1, 16, 17). There is, however, a lack of qualitative research on the child's response during a longitudinal disclosure intervention. The child's experiences are critical in gaining insight into the developmental appropriateness of the disclosure process, if it is supportive and well received, and how current disclosure practices can be improved.

There are various disclosure guidelines as well as there are theoretical frameworks of parental or caregiver HIV disclosure to children. Tasker's Four-Phase Model (FPM) describes disclosure as a process occurring in sequential phases, which is consistent with the South African National Department of Health [NDOH (SA)] disclosure guidelines as indicated in Table 1 (18, 19). Building on FPM, Blasini et al. (5) developed a structured clinical disclosure model for pediatric patients which incorporates five components for a positive disclosure procedure (5): (1) developmentally appropriate training of healthcare providers (HCP), (2) caregiver preparation for disclosure, (3) interactive sessions with the children, (4) full disclosure with the caregiver present, (5) post-disclosure follow up. This model postulates that these components will ensure better psychological adjustment for the child, increase the HCPs comfort with disclosure, and empower the caregiver to support the CHIV.

**Table 1:** A comparison between the stages of the four-phase model and the levels of disclosure described in the South African National Department of Health disclosure guidelines.

**Stages**	**Four-phase model**	**South African National Department of health levels of disclosure**
Stage 1	*Secrecy*: After learning of their child's diagnosis, caregivers feel shocked, lonely, and isolated. Fear and stigma result in caregivers keeping the diagnosis a secret and not telling the child, or even the family.	*Non-Disclosure:* The child is unaware of their illness and its effect on their body
Stage 2	*Exploratory:* Although caregivers continue to maintain the secret, they guard it with less intensity. They may provide explanations to the child, and may accurately describe HIV without naming it.	*Partial Disclosure:* The child is made aware of their illness without actually naming HIV
Stage 3	*Readiness:* The caregivers are becoming ready and begin to prepare to tell the child. This may mean actively seeking help from a healthcare provider.	*Full Disclosure:* The child is made aware of their illness which is named as HIV
Stage 4	*Disclosure:* The caregiver tells the child about their diagnosis, and may occur with or without the collaboration with a healthcare provider.	*Health Promoting Disclosure:* The child knows everything about their disease that is appropriate for their age. They are equipped in a supportive manner with skills to take age-appropriate responsibility for their health

We designed a study to quantitatively and qualitatively examine the outcomes of disclosure to CHIV in a longitudinal and developmentally appropriate manner. This paper reports on the qualitative outcomes and aims to describe the response of children during disclosure over time, as observed by the HCP facilitating the process. The disclosure process was structured based on a combination of various frameworks of HIV disclosure to CHIV, including recommendations by WHO and NDOH (SA) (18, 20) and encompassed the five components of Blasini's disclosure model (5). Our findings will contribute to knowledge that may help caregivers manage the CHIV after disclosure, as well as inform future interventions aimed at supporting children and their caregivers during and after the disclosure process.

## Materials and Methods

### Study Design

This was a longitudinal qualitative observational study that followed a cohort of CHIV through a gradual disclosure process over 78 weeks.

### Study Setting

Our study was conducted at the Perinatal HIV Research Unit (PHRU) located at the Chris Hani Baragwanath Hospital in Johannesburg, South Africa, between October 2017 and January 2020. The children resided in the greater Soweto area, a peri-urban township with a predominantly black population. The children were attending the pediatric HIV clinic or were enrolled in other active programs at PHRU, where they received HIV care and ART.

### Participants

Children who had not received full disclosure, as identified by the child's caregiver and their primary HCP at PHRU, were invited to participate. Informed consent was obtained from the caregiver (i.e., the child's biological parent, legal guardian, foster parent, or another person responsible for the protection and promotion of the child's wellbeing and development), and assent from the child. This purposeful sampling was used to screen 32 children, of which 30 were enrolled in this study. Children were included if they were between the ages 7 and 13 years, were able to comprehend the assent process, as well as communicate comfortably in a language of their choice. This age range was based on the recommendations by WHO and NDOH (SA) (18, 20). Exclusion criteria included: previous full disclosure as determined by the caregiver or HCP, known history of anxiety or depression, or an acute or medically significant event that would prevent participation in the study.

### Procedure

**Figure 1:**
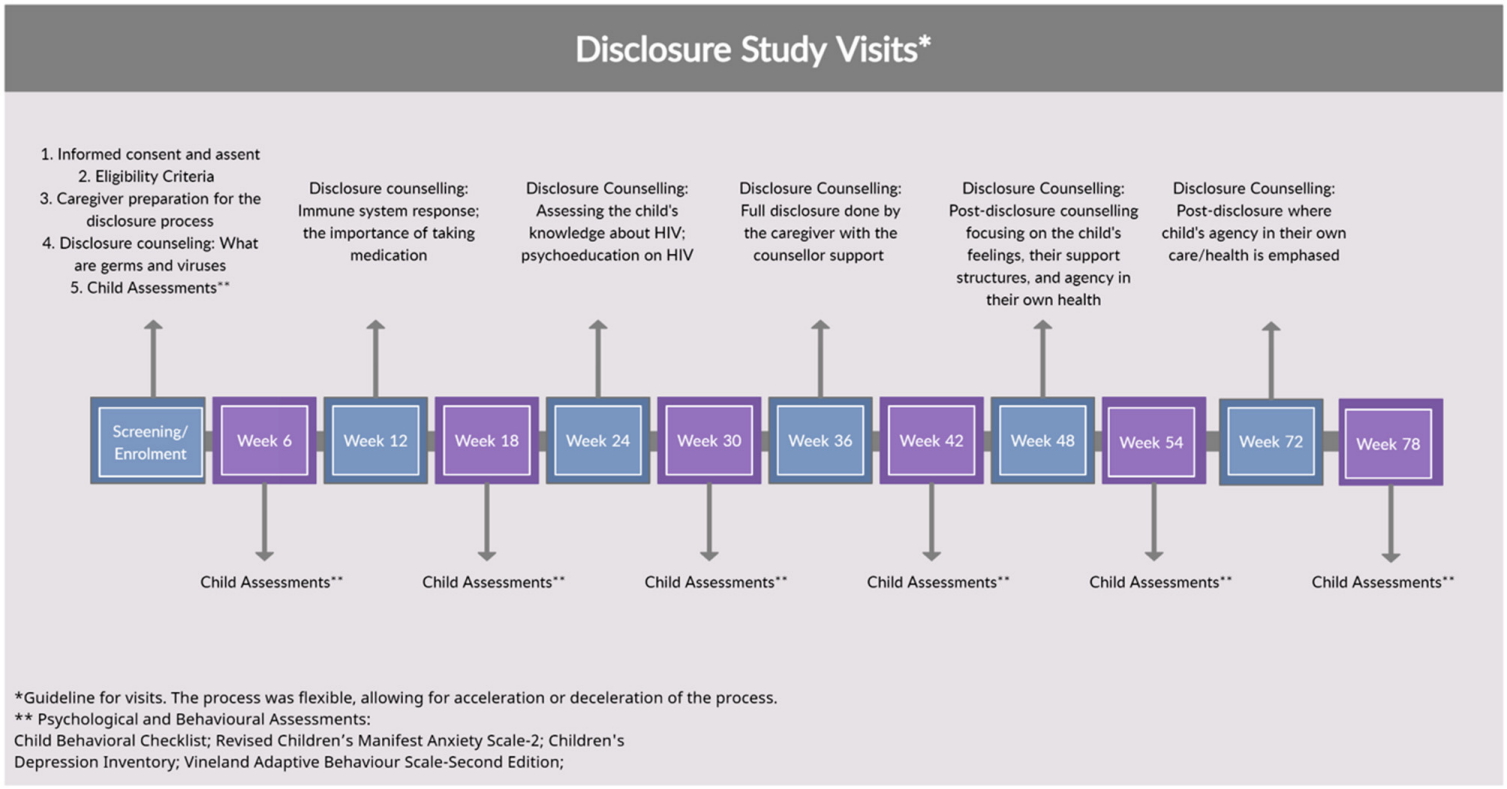
The disclosure study visit guideline for the 13 sessions conducted over 78-weeks, and what each visit entailed.

After signing informed consent and assent forms, the process began with a private counseling session with the caregiver. At this session they were counseled on what to expect during the disclosure process and how the sessions were structured, equipping them with the knowledge and skills for the full disclosure. The study ran over 78 weeks with ~13 study visits to accommodate enough time to reach the point of full disclosure and to allow an adequate time for post-disclosure observation. The study visits alternated between disclosure counseling sessions and psychometric assessment sessions as outlined in Figure 1. The 6 week intervals between visits was to assess the psychological distress triggered by the counseling sessions, and using the results from the psychometrics assessments to inform the next counseling session. The psychometric assessments included the Child Behavior Checklist, the Vineland Adaptive Behavior Scale, the Children's Depression Inventory, and the Revised Children's Manifest Anxiety Scale. The disclosure counseling sessions were based on the “Right-to-Care Mini Flipster” (21) and the United States Agency for International Development (USAID)/U.S. President's Emergency Plan for AIDS Relief (PEPFAR) disclosure booklets (22–24), which standardized the progression of disclosure over an average of six sessions. These counseling sessions progressed from partial, to full, to post-disclosure, where partial disclosure refers to making the child aware of illness without naming HIV. Full disclosure refers to making the child aware of their illness, naming HIV. Post-disclosure refers to counseling sessions after the child received full disclosure. The process was flexible and proceeded according to the child's comprehension of information, allowing for additional visits, revisiting of topics, and acceleration or deceleration of the process. The study visits were scheduled in addition to the child's regular clinic visits and where possible, effort was made for visits to coincide. Children's responses were documented and managed by trained HCPs. Following the study, those who were disclosed to at their last study visit were followed up and additional counseling was arranged where required. All participants continued to receive HIV care and ART at the PHRU pediatric clinic.

This paper focuses on the qualitative study that drew data from the six disclosure counseling sessions [the results of the psychometric assessment visits are described elsewhere (25)]. The counseling sessions were led by a female HCP, namely a counselor or a social worker, with both the caregiver and the child present, and were ~45 to 60 min long. The sessions were conducted in a language that the child was most comfortable communicating, primarily isiZulu and Sesotho. Both HCPs worked at PHRU and had extensive experience in pediatric HIV care and had received training on the disclosure tools used in this study. Directly after each disclosure counseling session, the HCP facilitating the session completed a counseling form documenting their observations and comments about the session including impressions of the participants, and the caregiver-child interaction. Using indirect observations, as opposed to interviews or focus groups, allowed HCPs to capture distress that children are not always aware of or able to express verbally.

### Counseling Forms

The paper-based counseling form was developed to document the disclosure progression in a consistent manner across participants. The form required HCPs to record the type of disclosure the child received in the session, namely partial disclosure, full disclosure, or post-disclosure counseling, as well as who accompanied the child. The topic of the day (germs, immune system, taking medication, HIV knowledge, and feelings about their status) was recorded along with noting whether the caregiver and child have had ongoing conversations about previous sessions. This often illustrated the caregiver-child relationship dynamics to the HCP. Progress notes indicated the topic to be covered at the next visit. Finally, the form required observations or impressions regarding the child during counseling session and any other relevant information that arose on the day, which may have included the caregiver-child dyad interaction or information on the child's adjustment outside of the study. The counseling form was completed in English directly after each counseling session using indirect observation and information provided by the caregiver and the child during the counseling discussions. HCPs were trained to complete these prompted questions, and record occurrences which they deemed notable during the counseling sessions. Their clinical backgrounds in pediatrics and training in disclosure equipped them with the necessary skills and experience to determine which behaviors to note. The HCPs were fluent in isiZulu and Sesotho (the languages primarily spoken by the caregivers and their children), as well as English. The counseling sessions were largely facilitated by HCP2, a counselor at PHRU, which reduced the variability in the way emotions were understood and recorded.

### Data Analysis

Each participant completed an average of seven counseling sessions. All seven forms per participant were analyzed manually using content analysis as per Erlingsson and Brysiewicz's guide (26). The counseling forms included those completed for additional sessions within the study visit schedule. Four analysts, with backgrounds in psychology, psychiatry and counseling, reviewed all the counseling forms. Each analyst independently hand-coded the data, and formulated categories by grouping codes of similar content or context. These codes and categories were compared across the analysts to formulate a single tabulation of categories, and discrepancies were discussed until consensus was reached. This tabulation was developed in Microsoft Word, with excerpts from the completed counseling forms quoted along with their corresponding codes and categories. Categories were then used to re-evaluate the data and to quantify the frequency of categories noted. Sub-themes were formed by clustering categories with shared underlying meaning. Lastly, the sub-themes were arranged into overarching themes.

Although content analysis relies on subjective interpretation, trustworthiness of data interpretation was improved by including four analysts with varying professional backgrounds and demographic characteristics, such as age and race. All analysts had qualifications which gave them experience with interpreting not only reported responses but also the subtle emotional nuances in the responses. None of the analysts were HCPs in the study, further reducing the likelihood of bias when interpreting the data.

### Ethics/Protection of Human Subjects

Ethical approval was obtained from the University of Witwatersrand Human Research Ethics Committee (HREC, Reference Number 170107), Johannesburg. Informed consent was obtained from all caregivers and assent was obtained from all child participants in the study.

## Results

### Participant Characteristics

Participant characteristics are summarized in Table 2. Of the 32 children approached, one failed screening due to comprehension and communication difficulties and the other knew their HIV status and was not eligible for the study. A total of 30 CHIV, with a median age of 10 years (IQR: 9.0–11.0) were enrolled, with 18 (60.00%) being female and participants pre-dominantly attending grades three to five. All children had at least one living parent, however, sessions were largely attended by mothers (26 mothers vs. 4 fathers). Of those, 28 parents were HIV-infected themselves and 27 used ART. Some sessions were also attended by maternal grandmothers and aunt for 3 participants. Most participants lived in formal housing (73%) and the median household crowding index was 1.67.

**Table 2:** Characteristics of children participating in the Disclosure study (*n* = 30).

**Variable**	**Overall**	**Male**	**Female**
Number of participants (%)	30 (100)	12 (40.00)	18 (60.00)
**Race**			
Black/African (%)	29 (96.67)	12 (40.00)	17 (94.44)
Mixed Race (%)	1 (3.33)	–	1 (5.56)
**Age**			
7–10 years (%)	16 (53.33)	5 (41.67)	11 (61.11)
11–12 years (%)	14 (46.67)	7 (58.33)	7 (38.89)
**Education level**			
Grade 2 (%)	2 (6.67)	1 (8.33)	1 (5.56)
Grade 3 (%)	7 (23.33)	3 (25.00)	4 (22.22)
Grade 4 (%)	7 (23.33)	3 (25.00)	4 (22.22)
Grade 5 (%)	8 (26.67)	3 (25.00)	5 (27.78)
Grade 6 (%)	4 (13.33)	1 (8.33)	3 (16.67)
Grade 7 (%)	2 (6.67)	1 (8.33)	1 (5.56)
**Caregiver relationship to child**			
Mother (%)	26 (86.67)	10 (83.33)	16 (88.89)
Father (%)	4 (13.33)	2 (16.67)	2 (11.11)
**Caregiver characteristics**			
HIV positive (%)	28 (93.33)	10 (83.33)	18 (100.00)
HIV positive caregivers on ART (%)	27 (96.43)	9 (75.00)	18 (100.00)
**Live in brick house (%)**	22 (73.33)	9 (69.23)	13 (76.47)
**Median (IQR) household**	1.67	1.75	1.25
**crowding index**	(1.00–2.50)	(1.00-3.00)	(1.00–2.33)

### Timing of Disclosure

Adherence to study visits was over 96.7%, with the majority of children (17/30, 56%) disclosed to at week 48, median [interquartile range (IQR)] age was 10.9 years (IQR: 10.5–11.3). Three children (10%) were able to receive full disclosure earlier than week 48 [age 10.2 years (IQR: 9.6–10.8)] and a quarter (7/30, 24%) were only ready to be disclosed to at the final visit [week 78, age 9.0 years (IQR: 8.6–10.2)]. One participant aged 7-years, completed all study visits but was not fully disclosed to due to poor comprehension. Two participants were no longer available for the study and their participation was discontinued, both participants not receiving full disclosure. The caregivers were no longer contactable after week 24 and week 48, respectively. The overall median age of children when receiving full disclosure was 11.6 years (IQR: 10.8–12.2).

### Key Categories

Three key categories are provided, with illustrative quotes from the written observations of the HCPs in Table 3.

**Table 3:** Identified categories and subthemes.

**Category**	**Description**	**Subtheme**	**Example quotes**
1. Emotions expressed by the children	Child's response through the disclosure process of partial, to full, to post-disclosure based on interactive sessions	Partial disclosure	“*The participant seemed anxious when we started the topic (HIV and modes of transmission) (100-003)*
		Full disclosure	“*The child displayed signs of being anxious, not wanting to talk about HIV” (100*-*012)* “*After finding out that he was HIV positive, he was very relaxed and seemed to talk better” (100*-*020)*
		Post disclosure	“*Child cried and hysterical uncontrollably when subject about her status came about” (100*-*029)* “*The participant is adjusting very well” (100*-*010)*
2. The development of the caregiver-child relationship	The progression of the relationship between the caregiver and child	Emotional	“*Mom tried to reassure [the child]” (100*-*028)*
		Behavioral	“*Mom and child have started bonding and there's a tremendous change in their relationship” (100*-*023)*
3. Cognitive appraisal of session content	The process of cognitive appropriation of the information given during the disclosure sessions	Comprehension	“*The participant struggled a bit to grasp the concept of germs” (100*-*014)* “*she was able to tie the last topic to today's topic”(100*-*011)*
		Engagement	”*The child wanted to read the story himself and he read well. Was very interesting to see the child realize that he need to eat better and also asked about the diet he's eating” (100*-*007)*
		Topic of HIV	“*The child was clued up on a lot of issues around HIV especially at her age” (100*-*010)* “*the participant was naïve for their age, she seems not to know anything about illness… the child does not seem to know anything about HIV” (100*-*002)*

#### Emotions Expressed by the Children

There were participants that displayed positive emotions during the partial disclosure sessions such as cheerfulness (3/30) and confidence (2/30), and were “*open to ask questions” (HCP2, female, 9 years old [y/o]). Seven* children were noted to have displayed negative emotions, particularly anxiety (5/30) and unhappiness (2/30), at one point during the study prior to the full disclosure session. Some children were nervous or uneasy during the first two sessions of the study. However, anxiety was often observed when the topics of HIV and HIV transmission were being addressed. The HCPs observed that four participants reported skin reactions/itchiness, feeling unwell and stomach aches when the topic of HIV was addressed.

“*the participant was very restless and started scratching themselves as we got deeper in our discussion claiming that his body was itchy”* (*HCP2, male, 11y/o)*.

The two participants that were unhappy were not perceived to be unhappy about attending the counseling session, but rather about events occurring in their lives, such as bullying at school and strained relationships with relatives. There were an additional seven participants who became more open and willing to communicate their emotions, as the sessions progressed through the study.

The HCPs observed that the 27 children who were fully disclosed to displayed an almost equal number of positive and negative responses during the session. 14 children responded to the disclosure with acceptance, often noting that they “*felt ok*,” with one participant verbalizing relief:

“*after disclosure, toward the end of the session, when he was asked how he was feeling, he said he's ok”* (*HCP2, male, 11 y/o)*.

The other 13 children were noted as having negative responses. The commonest response was crying (5/27), followed by discomfort (4/27), and then being “*shut off* ” (3/27) (*HCP2, female, 10y/o)*. The HCP noted that “*the session was very difficult, as the participant didnot want to talk after she was disclosed to” (HCP2, female, 10y/o). Two* participants were noted as passive during the session: “*participant was avoiding talking about HIV and she was very passive” (HCP2, female, 11y/o)*. Negative emotional responses included anxiety and avoidance (6/27), worry (3/27), sadness (3/27) and shock (2/27). Despite this, children who responded negatively were consolable, with observations of two children showing an improvement in distress by the end of the disclosure session, where “*in the beginning the child was reluctant to talk but eventually opened up”* (*HCP2, male, 10y/o)*. Similarly, while one child initially reacted to disclosure by withdrawing, this was transient and resolved by the end of the full disclosure session, “*She just needed assurance that she's still accepted and loved*” (*HCP2, female, 10y/o)*. One participant remained calm but was shocked about their status as they were under the impression they had another illness.

Post-disclosure counseling was done 12–24 weeks after full disclosure with 19 participants, each attending an average of 1–2 post-disclosure sessions. Six participants refused to speak during these sessions: “*when we started on HIV*+ *status, she became hysterical, crying and not wanting to talk*.”*(HCP2, female, 11 y/o)*. Four participants exhibited sadness or were upset during the post-disclosure session: “*Child was teary because of the fact that she is HIV* + *and that didnott sit well with her” (HCP2, female, 11 y/o)*, and an additional two participants required further reassurance:

“*she did express feelings of sadness and cried, but after reassuring her and talking about her feelings, she's much better” (HCP2, female, 10 y/o)*.

Ten participants were described as coping following full-disclosure, observed as making positive adjustments at home and school (10/19): “*participant started talking and she seemed fine, especially at school where she is excelling” (HCP2, female, 9 y/o)*. Six participants were perceived as being talkative and emotionally open during these sessions. One participant was observed as wanting to provide a “*good impression while not wanting to deal with real issues*” *(HCP2, female, 10 y/o)*.

#### The Development of the Caregiver-Child Relationship

7 caregivers and children were perceived to be emotionally closed off and not engaging during several sessions throughout the study: “*hope the mom and child will open up better” (HCP2, female, 10 y/o). 20* observed interactions were not considered to be close at the start of the study, with six caregivers being encouraged by the HCP to spend time with the child, “*Encouraged mom to bond more with her children” (HCP2, female, 10 y/0)*. 17 dyads developed closeness and bonded by the end of the process, and a “*tremendous change in their relationship”* was observed by the HCP *(HCP2, male, 10 y/o)*. Seven caregivers told the HCPs that they were close to their children, and openly communicated about various life topics. This was observed throughout the study.

Consistent ongoing communication and discussions about the session topics was found to be nearly equally present (9/30) and non-present (7/30) between the dyads. The remaining 11 dyads were inconsistent about reiterating the topics covered. The fluctuation was due to the caregivers work commitments or a different caregiver attending the session, such as the grandmother when the mother was unavailable. The topics covered were also observed as affecting the ongoing communication, with three dyads who avoided all communication around HIV:

“*Caregiver encouraged to talk about H.I.V issues and not ignore issues… it's a bit concerning that he never talked to his parents about his newly disclosed status” (HCP2, male, 10 y/o)*.

Of interest was that five children displayed discomfort with the caregiver present during the session, one child during the first session, and four other children after full disclosure,

“*participant asked the mom to step out… Mom and child seem close in their relationship. But it was still puzzling that she did not feel free to talk with mom in room” (HCP2, female, 10 y/o)*.

Another participant:

“*was a bit withdrawn though he asked for his grandma to step out a bit and asked if it is possible to be totally cured” (HCP2, male, 11 y/o)*.

#### Cognitive Appraisal of Session Content

During the initial visit, HCPs reported that two participants were “*confused about what has been said [during the session]” (HCP1, males, 8 and 11 y/o)*, while more than half of the participants [17/30, age 10.5 years (IQR: 9.2–10.9)] showed good comprehension of what germs and viruses are and how they make you sick. It was noted that caregivers reported that children learnt new things during the remaining sessions and strived to apply it at home, which was interpreted as the children retaining information from the sessions. Three children [age 8.9 years (IQR: 8.6–9.7)] were not ready to be disclosed to at the intended stage of the disclosure process since they were having difficulties remembering information from previous sessions. A total of six children [age 9.6 years (IQR: 8.9–10.2)] displayed poor comprehension of what they had learnt over the counseling sessions. *Five* children were described by the HCPs as being distracted and/or forgetful [age 10.9 years (IQR: 10.6–11.5)], and the HCP needed to spend more time than expected recapping the previous sessions.

The HCPs observed that on average, over half of the children [16/30, age 10.2 years (IQR: 9.6–11.0)] displayed behaviors described as quiet or avoidant of the session topics. The remaining 14 children were described as interactive, co-operative and actively participating in the sessions [14/30, age 10.7 years (IQR: 10.2–11.1)]. These children would answer the HCPs questions, give their opinion on the topics, and volunteer to read the material provided to them. They were also described as attentive, receptive and interested during the counseling sessions [6/30, age 10.0 years (IQR: 9.7–10.5)]. One child had overtly negative behavior, being “*very disruptive” (HCP1, male, 7 y/o)*, while *two* were noted as “*very restless..he would not sit still” (HCP2, male, 8 y/o)* and another two were described as being playful (*10 and 11 y/o*). However, one playful child was described as cheerful and participating well in the same session *(HCP2, female, 10 y/o)*.

Additionally, avoiding the topic of HIV was still evident in six participants [age 11.2 years (IQR: 10.7–11.5)] during post-disclosure as “*ignoring talking about HIV will make it disappear*” *(HCP2, male, 12 y/o)*. Furthermore, it was noted that one “*participant does not seem to associate herself with HIV”* (HCP2, female, 9 y/o). It was noted that five children [age 10.6 years (IQR: 9.9–11.4)] had suspicion of their illness: “*he said he suspected all along” (HCP2, male, 12 y/o)*.

Three were said to be curious and wanted to learn more about HIV [age 10.2 years (IQR: 9.5–10.8)].

“*He asked relevant questions about his status and also talked about his emotions… He is always asking questions, wanting to know more” (HCP2, male, 10 y/o)*.

## Discussion

Our findings show that children between the ages of seven and 13 were able to actively engage in the disclosure counseling sessions. However, as it might be expected, their responses varied when learning about their positive HIV status. A few children had negative reactions but were consolable, while others displayed an overall positive response as they engaged further, wanting more information on HIV and making positive adjustments to their lives. Furthermore, the relationship between the caregiver and child showed an overall improvement over the course of the study. There is a sparsity of literature describing the response of children to a disclosure process longitudinally, making this study unique in its insights.

Children's responses to the study changed over time depending on the stage of disclosure, and the content covered. While some approached the new process with anxiety, others were open and cheerful about the process. Our results show an increase in anxiety when the topic of HIV was addressed, and further distress when their own HIV status was disclosed. Negative responses such as sadness and worry are not uncommon and have been reported in a previous study (27), though based in retrospection which could potentially introduce bias. However, our results show that not all children reacted negatively to full disclosure. This is further supported by the psychometric assessments that were reported from the quantitative part of this study (25). The post-disclosure counseling sessions also indicated that although some children were having difficulty adjusting to their disease status, many were comforted, reassured, and were coping fine. The five components of Blasini's disclosure model (5) may guide key elements that moderate negative responses, namely training of HCPs, caregiver preparation, interactive sessions, full disclosure with caregiver present, and post-disclosure follow up. The interactive sessions are important in engaging the children, distinguishing their distress from inherent personality traits, and creating a trusting relationship with both their caregiver and HCP. These interactive sessions are also important in reiterating that HIV is not a death sentence, and correcting misinformation, thus contributing to reduced self-stigma, and in turn reduced distress following full disclosure. Our results show that those who became distressed often benefitted from reassurance from both the caregiver and the HCP, which was made possible by having the caregiver present during full disclosure. Post-disclosure sessions provide participants with a safe space to express their emotions and have them managed accordingly. The training component, coupled with HCP experience, enables the identification and management of behavioral manifestations of distress (externalizing and internalizing behaviors), as well as enabling appropriate support to the child and caregiver.

Children have diverse ways of expressing their emotions and use different coping mechanisms when processing distressing information. Depending on the child's developmental age and cognitive processing ability, they may express the magnitude and intensity of their feelings through their behavior (28). Children can exhibit externalized behaviors such as impulsivity, aggression and disruptiveness (29), described in our study as playful, disruptive, and somatizing anxiety prior to full disclosure, and openly crying when fully disclosed to. In contrast, children can also exhibit internalized behaviors characterized by anxiety, withdrawal and depressive symptoms (29), described in our study as quiet, anxious or avoidant, shut off and refusing to speak after being disclosed to, and becoming emotionally withdrawn, passive and unresponsive in later sessions. Distress can be reduced through allowing children to express their concerns and having their questions answered (27), providing them with accurate information and letting them know they are not alone (30), as well as through reassurance by the caregiver as shown in our results.

It is crucial to be able to identify and manage these expressions of emotional distress. Externalized behaviors are easier to identify due to the outward nature of expression. In our study, these behaviors may have been a reflection of the child's apprehension toward the study, anxiety induced by disclosure itself, or even a reflection of their caregiver's anxieties. This behavior may also be a result of the CHIV adjusting their “disease” status to their self-concept (5). Without the appropriate preparation as indicated in Blasini's model, caregivers may view children with externalizing behaviors as “naughty” or problematic, and overlook the child's distress or emotional struggle. This may lead to the later development of substance abuse disorder, risky sexual behaviors, relationship difficulties, financial difficulties, depression and anxiety (31). More care, however, needs to be taken with children who internalize their emotions, since their emotional distress may not be as apparent. Quiet and shy behaviors may also be identified as personality traits. Children that turn their emotions inwards may be more at risk of developing depression and anxiety later in life and/or having a poor self-image (32, 33). Our longitudinal approach to disclosure may also facilitate the identification and management of children's emotional distress. Caregivers can successfully be prepared to observe these internalizing behaviors, and refer to their HCP for further assistance (34).

A positive outcome of this study was the improved relationship and bond between the caregiver-child dyads. Many caregivers were observed as closed off at the beginning of the process, but as they progressed through the study and were encouraged to openly communicate about the session topics, improvements in relations was observed. This ensures that the child has a continued support structure, and encourages children to turn to their caregivers in times of need. Having the caregiver present during the sessions, particularly at full disclosure, allowed them to comfort and reassure the child. Often, children turn to their caregivers in times of need (35). However, for those few who did not, they may have been feeling distrust toward their caregiver as they had lied or hidden this truth from the child. They may have also not wanted to hurt their caregiver's feelings with their responses or questions. When disclosure is done in a clinic setting, the HCP, who is also a consistent figure throughout the process, plays an important role in comforting the child. This highlights the importance of the relationship with the HCP, establishing trust and creating a comfortable environment (14, 18, 20) as HCPs are also considered credible sources of information (36).

Post-disclosure sessions are essential as they ensure that no child and their distress is overlooked, particularly those who may internalize their emotions. It provides HCPs with an opportunity to follow up on changes in the child's engagement and behavior at home, school, and even with their friends. It also provides the child with a consistent and supportive environment in which they can express their emotions and work through their distress. Of note, when children did display emotional distress, it was not only in response to the counseling session itself but also stemmed from events happening in two of the children's lives. The caregiver preparation component may need to be emphasized, to allow for training on externalizing and internalizing behaviors, how to identify them, understanding what they mean, and how to manage it appropriately. This will empower caregivers in managing their children's distress and knowing when to seek additional help beyond the disclosure intervention.

Disclosure guidelines recommend partial disclosure to children as early as 3 years old (18). Our results indicated that children between the ages of seven and 12 were able to engage with the disclosure content by answering questions, providing their thoughts on the topics, and reading the material provided to them. Many of the children were also able to understand the content of germs, their immune system and HIV. This is contrary to what caregivers and HCPs have reported as a barrier to disclosure (37, 38). Those that were quiet during the sessions may be a result of the disclosure counseling being pitched at a level that was not compatible with the child's developmental stage, particularly younger children. This was seen in the youngest participant of 7 years old who struggled to grasp the concepts and was not fully disclosed to. Cognitive development is not always equivalent to chronological age, and counseling should be contextualized to each child's cognitive development, which would then guide when and how new information should be shared (34). This is advocated for in Blasini's model, as well as the NDOH (SA) guidelines (5, 18). Our results also indicate a range of age-related responses. Children up to the age of 9 years may require more time or additional sessions to reach full disclosure. Those that were 10 years old at full disclosure had a range of responses, from crying and being passive to being attentive and accepting, while children aged 11 and older were more avoidant of the topic of HIV, even during post-disclosure sessions. Given this range of responses, a longitudinal approach is best to begin partial disclosure with younger children and progress to full disclosure. Those that start the process at an older age would require multiple post-disclosure counseling, particularly those who display negative responses and avoidant behavior.

The emotional, relational and cognitive progression described above are not independent of each other. The emotional response of the child were sometimes dependent on the topics covered. Reiterating the topics, and ensuring it was well understood may mitigate extreme negative responses. The on-going communication on the session topics not only facilitated the cognitive appropriation of the content, but also encouraged frequent communication between the caregiver and child, which in turn improves the relationship. Improvement in the relationship allowed caregivers to reassure children following full disclosure. Having the caregiver present for the sessions also meant that the caregiver could learn how to communicate about HIV, and they may have also clarified misconceptions that the caregiver had and alleviated their anxiety. With the caregiver at ease, this could further comfort the child, easing their emotional distress. This is an important cyclic process that can be achieved with a longitudinal approach to disclosure.

This study was not without its shortcomings. Although the sample size was small, we did follow our sample longitudinally and maintained above 90% sample retention. This high retention may be due to the long-standing relationship with the PHRU staff and clinic. One of this study's limitations is that not all the nuanced details of the disclosure counseling sessions were reported. Future studies should capture detailed reports of the participants' experience for a better understanding of their emotional response and processing of the disclosure. There should also be several follow-up or post-disclosure sessions to ensure distress is identified and adequately managed. Our study is also based on the observations of the HCP, which may be biased and does not accurately capture the child or caregiver's perceptions, feelings, and views. However, the HCP was able to observe behaviors that indicated distress, which the child and caregiver may not always be aware of, or willing to acknowledge. The study design was lengthy and would be difficult to implement in local clinics where HCP's may not be as familiar with all patients. Due to the long-standing relationship between participants and PHRU healthcare providers, participants may have felt secure and supported, and this may have alleviated potential negative responses. Alternatively, less negative responses may have been seen as caregivers and children may have only reported and displayed behaviors that they perceived as socially favorable. However, the longitudinal approach of our study may mitigate social desirability bias as trust is built with the HCPs. The applicability of Blasini's disclosure model is uncertain as it was designed and tested on a Puerto Rican sample. However, we found that it was a good fit with our participants, possibly due to the similarities in age. This speaks to the applicability of the model across various cultures and demographic characteristics when designing future disclosure interventions.

## Conclusion

This paper has described the complexity in children's responses to disclosure as observed by healthcare providers. Importantly, our results show that those who do become distressed often benefitted from reassurance during and post-disclosure counseling, being better able to process negative emotions and behavior by the end of the disclosure session. Blasini's disclosure model highlighted the elements that may contribute to moderating negative responses. Future interventions should emphasize training on identifying, addressing and managing children's internalizing and externalizing behavioral manifestations of distress, as it is critical in providing them with the necessary support they need during and after disclosure. Having this guidance for disclosure intervention development may alleviate HCP and caregiver anxiety around disclosure, thus not only improving rates of disclosure, but also fostering a more positive disclosure experience for the child.

## Data Availability Statement

The raw data supporting the conclusions of this article will be made available by the authors, without undue reservation.

## Ethics Statement

The studies involving human participants were reviewed and approved by University of Witwatersrand Human Research Ethics Committee HREC (Reference Number 170107). Written informed consent to participate in this study was provided by the participants' legal guardian/next of kin.

## Author Contributions

AL, CJ, JB, AV, and KO contributed to the study conception and design. Material preparation, data collection, and analysis were performed by GL, AL, CJ, LG, and CR. The first draft of the manuscript was written by CR. All authors commented on previous versions of the manuscript. All authors read and approved the final manuscript.

## Funding

This study was funded by the South African Medical Research Council's extramural funding to the Perinatal HIV Research Unit (PHRU).

## Conflict of Interest

The authors declare that the research was conducted in the absence of any commercial or financial relationships that could be construed as a potential conflict of interest.

## Publisher's Note

All claims expressed in this article are solely those of the authors and do not necessarily represent those of their affiliated organizations, or those of the publisher, the editors and the reviewers. Any product that may be evaluated in this article, or claim that may be made by its manufacturer, is not guaranteed or endorsed by the publisher.
