# Supplementary material for: Children's Positive and Negative Emotional Responses to an HIV Disclosure Study in South Africa
Source: Front Pediatr. 2022 May 31;10:857336. doi: 10.3389/fped.2022.857336 (PMC9192950; doi:10.3389/fped.2022.857336)
Supplement: Supplementary file 1 [file Data_Sheet_1.PDF]

DISCLOSURE COUNSELLING FORM

Disclosure Study

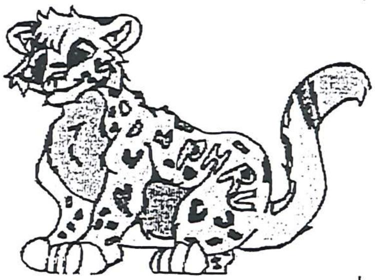

Participant's PID number: ____________________

Visit Number: ________________

Date: ________________

1. Type of disclosure done today (Please circle the applicable option below):

Partial	Full	Post-disclosure

2. If this is the first encounter of the disclosure process, which factors that formerly prevented the caregiver from disclosing to the child? (If not, proceed to next question)

____

____

____

____

3. Why was this type of disclosure done today?

____

____

____

4. What was discussed today:

____

____

____

____

5. Who accompanied the child to this disclosure?

____

6. Has the caregiver and the child had ongoing conversations about what was discussed previously? (Skip if this is the first encounter)

____

____

____

7. What topics will be covered at the next visit?

____

____

____

8. What was your impression of the disclosure counselling session?

____

____

____

9. Other comments: ____

____

Signature of person completing the form:	*Name of person completing the form*	*Date:*

Version 1.0; 04 October 2017

Supplementary document 1: Disclosure counselling form developed for the PHRU disclosure study to document the disclosure progression in a consistent manner across participants
